# Altered Oscillatory Dynamics of CA1 Parvalbumin Basket Cells during Theta–Gamma Rhythmopathies of Temporal Lobe Epilepsy

**DOI:** 10.1523/ENEURO.0284-16.2016

**Published:** 2016-11-18

**Authors:** Diego Lopez-Pigozzi, François Laurent, Jorge R. Brotons-Mas, Mario Valderrama, Manuel Valero, Ivan Fernandez-Lamo, Elena Cid, Daniel Gomez-Dominguez, Beatriz Gal, Liset Menendez de la Prida

**Affiliations:** 1 Instituto Cajal, Consejo Superior de Investigaciones Científicas, 28002 Madrid, Spain; 2 Departamento de Ingeniería Biomédica, Universidad de los Andes, 111711 Bogotá, Colombia; 3 Villaviciosa de Odón Campus, Universidad Europea de Madrid, 28670 Madrid, Spain

**Keywords:** episodic memory, hippocampus, silicon probes, tetrodes, theta-gamma

## Abstract

Recent reports in human demonstrate a role of theta–gamma coupling in memory for spatial episodes and a lack of coupling in people experiencing temporal lobe epilepsy, but the mechanisms are unknown. Using multisite silicon probe recordings of epileptic rats engaged in episodic-like object recognition tasks, we sought to evaluate the role of theta–gamma coupling in the absence of epileptiform activities. Our data reveal a specific association between theta–gamma (30–60 Hz) coupling at the proximal stratum radiatum of CA1 and spatial memory deficits. We targeted the microcircuit mechanisms with a novel approach to identify putative interneuronal types in tetrode recordings (parvalbumin basket cells in particular) and validated classification criteria in the epileptic context with neurochemical identification of intracellularly recorded cells. In epileptic rats, putative parvalbumin basket cells fired poorly modulated at the falling theta phase, consistent with weaker inputs from Schaffer collaterals and attenuated gamma oscillations, as evaluated by theta-phase decomposition of current–source density signals. We propose that theta–gamma interneuronal rhythmopathies of the temporal lobe are intimately related to episodic memory dysfunction in this condition.

## Visual Abstract

**Figure:**
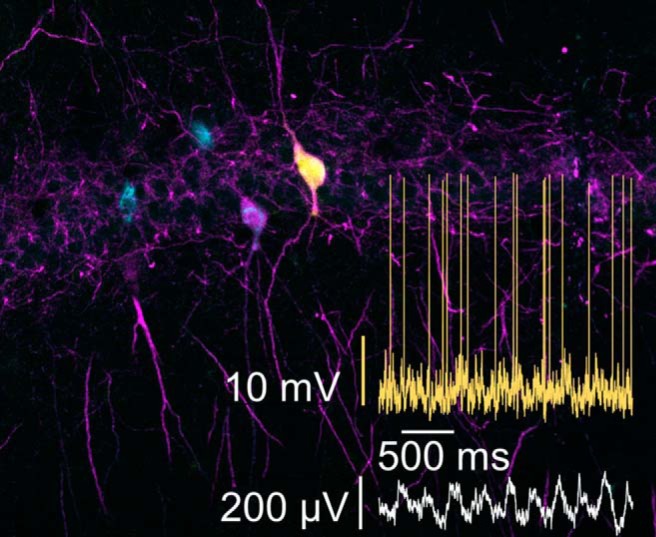

## Significance Statement

Cognitive deficits of temporal lobe epilepsy represent a major comorbidity of this disease, but how epileptogenic microcircuits serve cognitive demands during seizure free periods remain poorly understood. Here, we investigate this issue using an experimental rat model of temporal lobe epilepsy. Our data link hippocampal theta phase-specific uncoupling in the slow gamma band with disruption of episodic-like memory function and microcircuit deficits resulting from poor oscillatory firing of parvalbumin basket cells.

## Introduction

Interaction between brain oscillators has been revealed as a critical mechanism to orchestrate synchronization between neuronal circuits (Varela et al., 2001). One example is theta–gamma coupling, a phenomenon by which the phase of hippocampal theta activity (4–12 Hz) modulates the amplitude of gamma activity (30–120 Hz; Canolty et al., 2006). Theta–gamma coupling can be expressed locally in hippocampal microcircuits (Soltesz and Deschênes 1993; Bragin et al., 1995; Scheffer-Teixeira et al., 2012; Schomburg et al., 2014), and distantly to control the interaction with hippocampal targets, such as the prefrontal cortex (Mitchell et al., 2008). It is believed that theta-nested gamma oscillations provide an efficient code for neuronal processing on demand (Montgomery and Buzsáki, 2007; Colgin et al., 2009; Mizuseki et al., 2009; Tort et al., 2009; Bieri et al., 2014; Cabral et al., 2014)

In the hippocampus, different gamma generators couple preferentially to distinct theta phases. Mid-gamma generator (60–90 Hz) operates at the peak of the CA1 hippocampal theta oscillation recorded at the stratum pyramidale (SP; Schomburg et al., 2014) in coordination with the arrival of entorhinal layer III temporoammonic inputs into the stratum lacunosum moleculare (SLM; Mizuseki et al., 2009). An intrahippocampal slow gamma generator (30–60 Hz) operates at the falling phase of theta coincident with CA3 Schaffer inputs arriving at the stratum radiatum (SR; Csicsvari et al., 2003; Lasztóczi and Klausberger, 2014). A third, even faster, generator (>100 Hz) runs locally at SP during the negative peak of theta when CA1 pyramidal cells (PCs) fire maximally (Schomburg et al., 2014; Butler et al., 2016). A detailed proximodistal arrangement along the hippocampal axis from CA3 to subiculum implements entorhinal–hippocampal function (Henriksen et al., 2010; Bellistri et al., 2013; Schomburg et al., 2014; Laurent et al., 2015). Presumably, the unique role of hippocampus in episodic memory relies in such ability to segregate/bind activity from different communication channels while encoding and retrieving information about events.

Temporal lobe epilepsy (TLE), a disease affecting the hippocampus and parahippocampal regions, is associated with a range of cognitive comorbidities, and episodic memory deficits in particular (Helmstaedter 2002). Although investigating the mechanisms of epileptogenesis and ictogenesis draw attention, we still lack integrative theories to understand a range of cognitive comorbidities affecting this disease. Strikingly, if one considers the ictal dynamics across the temporal lobe, seizures seize the same microcircuits responsible for rhythmogenesis, suggesting shared grounds. For instance, dysfunction of the temporoammonic pathway has been related both to seizures (Wozny et al., 2005; Ang et al., 2006) and to chronic theta rhythmopathies affecting memory function in TLE (Dugladze et al., 2007; Inostroza et al., 2013; Laurent et al., 2015). Recently, it has been suggested that there is a critical role for theta–gamma coupling in episodic memory formation in healthy people and a lack of such a role in the epileptogenic hippocampus (Shirvalkar et al., 2010; Lega et al., 2015, 2016). Whether the microcircuit dysfunction underlying epileptogenesis is related to theta–gamma rhythmopathies and the associated cognitive deficits remains unknown.

Here, we demonstrate that disruption of theta–gamma coordination in the dorsal hippocampus of TLE rats is linked to specific aspects of episodic-like memory deficits in these animals in periods free of seizures or interictal activities. We elucidated the mechanisms of this phenomenon with a combination of multisite and single-cell recordings, and found that dysfunction of parvalbumin (PV) basket cells, a resilient interneuronal population in TLE, may be central to this form of hippocampal rhythmopathy.

## Materials and Methods

All experimental protocols and procedures were performed according to the Spanish legislation (R.D. 1201/2005 and L.32/2007), and the European Communities Council Directives of 1986 (86/609/EEC) and 2003 (2003/65/CE) for animal research, and were approved by the Ethics Committee of the Instituto Cajal and the Spanish National Research Council.

### TLE model

Adult male Wistar rats (180–200 g) were treated with multiple intraperitoneal injections of kainate (5 mg/kg) at hourly intervals until they reached status epilepticus. Diazepam (4 mg/kg) was injected 1 h later to stop convulsions. Animals received intraperitoneal injections of 2.5 ml of 5% dextrose in saline, and their diet was supplemented with fruit and powder milk during the following 2–3 d. After 4–5 d post-status, rats behaved normally and were housed individually. Some rats were injected with saline instead of kainate and received treatments similar to those in epileptic animals. Untreated normal rats completed the control group, since no differences between them and saline-injected animals were detected in electrophysiological indices. All quantitative experiments started 8 weeks after status when rats already exhibited recurrent seizures being in the chronic phase. Seizures were either observed during animal handling or recorded electrophysiologically during behavioral tasks. In epileptic rats, periods of normal-like electrophysiological activity were intermixed with periods dominated by epileptiform events, defined as convulsive or subclinical seizures and interictal discharges (Inostroza et al., 2013). Only data from sessions free from any sign of epileptiform activity within 2 h were considered for the analysis of hippocampal oscillations.

A total of 18 normal rats (12 untreated, 6 vehicle treated) and 17 epileptic rats were used in the study.

### Chronic electrophysiology

To implant either multisite silicon probes or tetrodes, rats were anesthetized with isoflurane (1.5–2%) in oxygen (30%) and were continuously monitored with an oximeter (MouseOx, Starr Life Sciences). Epileptic and saline-treated rats were implanted 6–7 weeks after treatment to allow for at least 1 week of recovery and stabilization before quantitative experiments started. Implantation coordinates ranged from 3.9 to 6 mm posterior to bregma, and between 2 and 5 mm from midline. Sixteen-channel silicon probes (NeuroNexus; site impedance, 0.3–1.2 MΩ; resolution, 100 µm; electrode area, 413 µm^2^) were implanted either fixed or mounted on an adjustable microdrive (either custom-made or the nDrive from NeuroNexus). For tetrode recordings, microdrives (VersaDrive, Axona) with eight independent screws loaded with tetrodes (13 µm platinum-iridium wires, California Fine Wire) were used. All electrodes were implanted perpendicular to the skull surface. Two screws served as a reference and ground at the occipital region. After surgery, rats received short-term treatment of enrofloxacin 10 mg/kg, methylprednisolone 10 mg/kg, and buprenorphine 0.05 mg/kg.

After recovering, electrophysiological recordings were obtained using a multichannel amplifier (Dacq System, Axona). Local field potential (LFP) signals from multisite silicon probes were acquired with a 16-channel headstage, amplified by 400 and stored at 4800 Hz/24 bit precision in a 1–2400 Hz frequency band after analog filter. Signals from tetrodes were obtained with 32-channel headstages, amplified (400× to 1000×), bandpass filtered (0.3 Hz to 24 kHz; Dacq System, Axona), and stored at 24 kHz/24 bit precision. In all cases, the position of the rat in the arena (*x*, *y*) was estimated from an infrared LED coupled to the headstage and imaged from a ceiling camera (100 Hz, 300 pixels/m).

All electrophysiological recordings began 3–7 d after surgery when animals had already recovered. For multisite probes, we checked that indices of theta power and coherence between layers were stable (<10% variability) over consecutive days for a given animal before starting experiments. In animals carrying microdrives, the position of the silicon probe was adjusted in depth to target similar layers over different days. For tetrodes, screening was performed to check for the ability to record units and to infer the electrode position by lowering each tetrode independently toward the hippocampus. Every day, single tetrodes were advanced individually until unit-firing and recognizable hippocampal activities [sharp-wave ripples (SPWs), high-amplitude theta] were identified. Recordings were obtained daily from Monday to Friday between 2:00 and 7:00 P.M. over 1–3 weeks while the animals performed different cognitive tasks.

We implanted silicon probes in 10 control rats (4 untreated, 6 vehicle treated) and 10 epileptic rats. After completing experiments, some of these rats were recorded in the dead condition (after overdose of anesthesia) with the silicon probe in place before fixation, to evaluate aspects of the 1/*f* spectral behavior (*n* = 2 untreated control rats, *n* = 2 epileptic rats). For tetrode recordings, three control rats (untreated) and three epileptic rats were used.

### Object recognition tasks

We used the “what-where-when” paradigm of object recognition tasks to test for episodic-like memory in TLE rats (Inostroza et al., 2013). The test apparatus consisted of a square open field (80 × 80 × 50 cm) situated in an evenly illuminated room (15 l×) with ambient noise masked by white noise and several cues visible on the surrounding walls. Animal behavior was monitored with a video camera, and exploration was analyzed off-line either with EthoVision (version 1.90, Noldus IT) or by routines written in MATLAB. Odor cues were removed after each trial with 0.1% acetic acid. Before the task, animals were habituated over 3–4 d to open-field exploration and LFP recordings, to get them familiar with the tethered system.

During the task, animals encountered four copies of an object during a first sample phase (old familiar objects A, 3 min duration). Objects were chosen after confirming no specific preferences, and that they were big enough to avoid climbing or dropping (10–15 cm tall, 8–10 cm base). A second sample phase (3 min duration) followed 50 min after the first, in which rats found four copies of another object (recent familiar objects B; see Fig. 4*A*, scheme). The second objects occupied either a new location or the same location as the objects seen before. The test phase (3 min duration, 50 min after the second sample phase) consisted of exposing rats to two old and two recently familiar objects seen in the previous sampling phases. During the intertrial period (50 min), animals were left in a home cage to which they had previously been habituated. Rats entered the arena from a fixed position, randomized between animals.

Control rats exhibited strong exploratory preference for the old familiar stationary object (A1), presumably reflecting a temporal component of episodic-like memory, and for the recent familiar displaced object (B2), reflecting spatial memory for events (Kart-Teke et al., 2006). The what-where-when task, together with the “what-where-which” task (Eacott and Easton, 2010) and other single-trial object recognition tasks (Ennaceur and Delacour, 1988) are considered valid cognitive tests to evaluate episodic-like memory abilities in rodents. Electroencephalographic recordings were obtained continuously during the different phases of the task to ensure that epileptiform activities were identified.

To quantify performance in the task, a discrimination index was estimated for every object by dividing the time spent at each object by the total exploratory time and tested against chance level (0.25; one-sample *t* test). We also defined an index of the spatial memory component (the “where” index) as the proportion of time spent exploring the displaced object B2 against the other stationary object seen in sample phase 2 divided by the total time spent with them (Inostroza et al., 2013; see Fig. 4*A*, scheme). Similarly, an index of the temporal memory component (the “when” index) was defined as the proportion of time spent exploring the old stationary object A1 seen in sample phase 1 against the recent stationary object seen in sample phase 2 divided by the time spent exploring them together (for details, see Inostroza et al., 2013). For these indices, a value of zero indicates no preference (chance level).

We also used a one-sample object recognition task to test for spatial recognition memory (where) independently (Ennaceur and Delacour, 1988; Inostroza et al., 2013). In this task, two identical objects were used in the sample phase (3 min). The rat exploratory preference for a displaced object was tested in a test phase (3 min duration) 50 or 100 min after. Animals entered the arena allocentrically between phases. A discrimination index was calculated as the ratio between the time spent with the displaced object and the total exploratory time.

For these experiments, six control rats (four untreated, two vehicle treated) and six epileptic rats implanted with silicon probes were tested in the what-where-when task. We also tested four control rats (two untreated, two vehicle treated) and four epileptic rats in the one sample where task. Two epileptic rats and one control rat were tested in both tasks. In addition, for rats implanted with tetrodes, we ran the what-where-when task in one control rat (untreated) and one epileptic rat, and the where task in two control rats (untreated) and two epileptic rats. Remarkably, all these epileptic rats were free of epileptiform activities within 2 h of the tasks. In four epileptic rats, seizures and/or interictal activities were recorded during the what-where-when task, and they were analyzed separately. In three of these animals, episodic-like memory was retested on a different day in the absence of any sign of epileptiform activities. All memory tasks were performed at equivalent times of day.

### Open-field exploration and pellet-chasing tasks

We used sessions of free exploration of different arenas without objects (50 × 25, 50 × 50, and 80 × 80 cm; all 50 cm tall) to screen hippocampal layers and units. These sessions were intermixed with the object recognition tasks, either as habituation sessions for the task itself or as independent screening sessions. After completing object recognition tasks, we ran standard pellet-chasing sessions to improve unit sampling from tetrodes and to eventually establish their place cell behavior. To this purpose, rats implanted with tetrodes were deprived of food for up to 85–90% of their original weight. Initially, animals were allowed to explore freely the open field over 3–5 min. During each session (10–15 min), small pellets of food dropped in every 20 s to random locations within the open field, keeping the animal in continuous locomotion and allowing a full sampling of the arena. Electroencephalographic recordings were obtained continuously during free exploratory and pellet-chasing sessions.

All rats implanted with tetrodes were tested with these tasks (three untreated control rats, three epileptic rats). Some rats implanted with silicon probes were tested with pellet chasing, but data are not discussed (one untreated control rat, one epileptic rat).

### Spectral analysis

Signals were analyzed using routines written in MATLAB (MathWorks version 10b) and/or Octave (version 3.6.4; www.octave.org). One session (300 s) free of artifacts (mostly due to interactions between headstage and objects during the exploration of the rat) and with >14 functional channels spanning around SR and SLM were selected from the analysis of multisite LFPs from each animal. Current source density (CSD) signals were calculated using the second spatial derivative of LFPs at 100 µm resolution. Smoothing and defective site interpolation were used for visualization purposes only. CSD signals from the SP layer could not be consistently evaluated since they were typically sampled by the first recording channel or by none. LFP signals from tetrodes were analyzed similarly.

**Figure 1.**
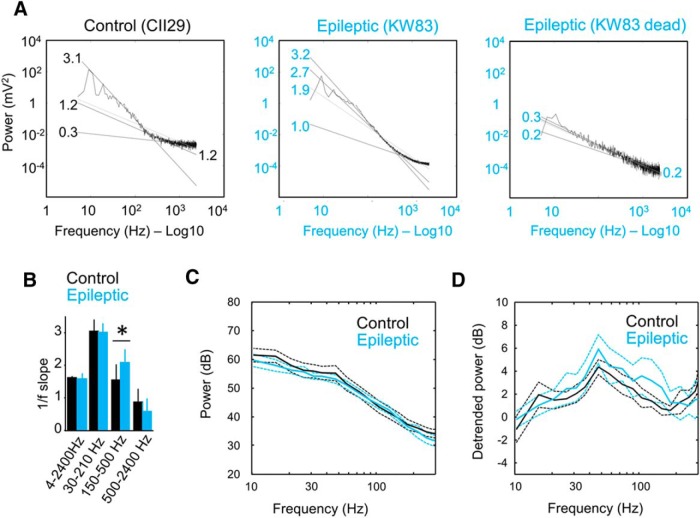
Correction for 1/*f^n^* to evaluate group differences across different gamma bands. ***A***, The typical 1/*f* trend of the power spectrum complicates the examination of a range of gamma activity from 30 to 150 Hz. Representative spectrum from LFP at the SLM from a control and an epileptic rat is shown to exemplify the issue. The codes of the rats are given in parentheses. The factor *n* better fits the 1/*f* power law change for different frequency bands. Numbers at fitting lines identify the *n* factor for different bands in the examples shown (4–2400, 30–210, 150–500, and 500–2400 Hz). Note the fitting consistency between bands when no biological activity is recorded (dead condition for the epileptic rat; rightmost plot). ***B***, Group differences of the *n* scale factor for different frequency bands. Note the similarities between control and epileptic rats, except for the 150–500 Hz high-frequency band. *p < 0.05. ***C***, Mean group power spectrum (±95% confidence interval) from SLM channel of all control rats (*n* = 10) and epileptic rats (*n* = 8). Note the occurrence of some spectral trends across gamma bands but poor difference between groups due to 1/*f* scaling. ***D***, Same data as in ***C*** after power law detrending between 30 and 210 Hz. Note the differences between groups.

**Figure 2.**
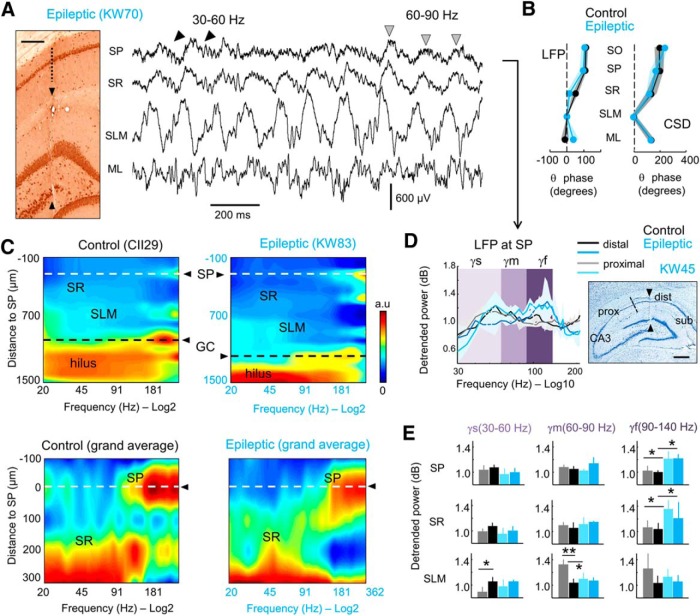
Different types of gamma activity recorded in the dorsal hippocampus of epileptic rats. ***A***, Representative example of an epileptic rat recorded with 16-channel silicon probes during object exploration. Left, Coronal section immunostained against NeuN shows the probe track. Scale bar, 200 µm. Right, Representative LFP signals. Note gamma activity along the descending phase of some theta cycles at the SP (trace: 30–60 Hz, black arrowheads). Mid-gamma activity (60–90 Hz) is typically recorded at the positive theta peak of some cycles (gray arrowheads). SR, Stratum radiatum; ML, molecular layer of the dentate gyrus. ***B***, Similar laminar phase shift of LFP and CSD theta cycles with respect to SLM (mean ± 95% confidence interval; *n* = 10 control, *n* = 8 epileptic). ***C***, Spatial expression of gamma activity across hippocampal layers in a representative control and epileptic rat (top plot). Note the stereotyped profiles characterized by stronger activity at the hilus and distinct gamma bands at different layers. To avoid the hilar saturating effect, a grand average of three control and three epileptic rats recorded at comparable locations is shown at bottom along the SP and SR layers. ***D***, Relative power after 1/*f* detrending from LFP signals recorded at the CA1 SP (mean ± 95% confidence interval). Data are grouped according to proximal (close to CA3) and distal [close to subiculum (sub)] recording locations (see scheme at right). The three gamma bands are indicated by different colors: gamma-s (γs; 30–60 Hz; light purple); gamma-m (γm; 60–90 Hz; purple); and gamma-f (γf; 90–140 Hz; dark purple). Right, Nissl staining section showing proximal and distal locations along with the probe track (arrowheads). Scale bar, 500 µm. ***E***, Group differences on the three gammas for LFP recorded at SP, SR, and SLM from all rats. Data (mean ± SD) are presented as proximal (*n* = 5 control rats; *n* = 4 epileptic rats) and distal (*n* = 5 control rats; *n* = 4 epileptic rats) for SR and SLM layers. Note strong statistical differences accumulated at the fast gamma band. *p < 0.05; **p < 0.005

Data segments (1 s, nonoverlapping) with continuous theta were identified from different recording sessions (300 s each) using spectral criteria, as previously described (Laurent et al., 2015). Control and epileptic animals expended similar lengths of time exploring objects and the open field, resulting in comparable epochs of exploratory theta activity (Inostroza et al., 2013, their Fig. 5). For analysis of the entire frequency band (1–2400 Hz), a Hamming window and the fast Fourier transform (FFT) at 1 Hz resolution were used. For analysis of gamma activity, spectral power was estimated from the 5-Hz-step FFT from 30 to 250 Hz. The contributions of 50 Hz and harmonics were filtered out, and data between the filter limits were interpolated. To overcome the effect of decay across gamma bands, power spectra were detrended (Fig. 1), similar to prewhitening but without fitting to an autoregressive model that may be either too rigid to capture the multicomponent 1/*f* or too flexible in removing part of the spectral structure. We found approximately similar mean spectral indices across different exploratory sessions (habituation, open-field, sample, and test phases of object exploration) for each rat, suggesting that they capture basal spectral features of hippocampal microcircuits (Inostroza et al., 2013). For visualization of the full gamma band across layers (Fig. 2*C*), power spectra were estimated with a Gabor wavelet.

The positive peak of theta waves at SP was used as a reference to study theta–gamma modulation using phase-decomposition approaches (Schomburg et al., 2014). To estimate theta peaks accurately, we took advantage of stronger theta oscillations at SLM in multisite recordings. Thus, we first filtered LFP signals from SLM between 4 and 12 Hz to detect negative theta peaks, which were then realigned using the 1–100 Hz (50 Hz excluded) filtered signal. We next identified the associated positive theta peak at SP whenever the time lapse between two successive troughs corresponded to an instantaneous frequency in the 6–10 Hz frequency range. Realignment using the wider band-filtered signal was critical to avoid differences of theta wave asymmetry between groups, confounding the analysis of theta–gamma interaction. In three rats where the SP layer was not sampled, the theta peak was inferred by extrapolation cycle by cycle (Fig. 2*B*, phase-reversal curve). The accuracy of this approach was confirmed in rats with SP recordings (estimated phase error at SP, −0.86 ± 0.76º). Tetrode data were analyzed similarly, but only theta peaks were detected at the cell body layer. Theta peaks and troughs were defined as one peak between two successive previously detected troughs and one trough between two successive previously detected peaks, respectively. Filtering and peak/trough detection were performed with a sliding window (1 s, 50% overlap). The timestamps of peaks and troughs were matched to define a single time series for the entire session. For each theta cycle, a peak-centered segment was defined with duration equal to 2.2 times the average cycle duration in the session. This allowed accounting for the slight difference in theta frequencies between control and epileptic rats (Inostroza et al., 2013).

To evaluate theta-phase coupling of gamma activity, the following phases were defined with respect to the theta peak at SPk, as follows: −2π, −3/2π, −π, −π/2, 0, π/2, π, 3/2π, and 2π. Phase segments were defined based on the average cycle duration. The FFT was calculated for each phase segment, and detrended in each session considering all of the available segments. The 1/f behaviour of LFP spectrum led us to make use of these detrended Fourier coefficients instead of other phase-amplitude measurements (Canolty et al., 2006). Data from the first and last phases were discarded to avoid border effects. Detrended gamma power was estimated from phase segments in various frequency ranges, as follows: slow gamma (gamma-s) values, 30 − 60 Hz; mid-gamma (gamma-m) values, 60 − 90 Hz; and gamma-fast (gamma-f) values, 90 − 140 Hz (previously validated by source separation with independent-component analysis; Schomburg et al., 2014).

A modulation index was defined for each gamma band by estimating the difference between the maximal and the minimal gamma power modulated across the theta cycle using data from −π to π. To test the modulation index statistically, we chose a conservative surrogate design that destroyed phase-specific gamma coupling while preserving unspecific nonstationarities of the original data. In preliminary tests, we noted that phase segment shuffling resulted in equalized gamma activity across the theta cycle. To avoid this effect, we cut the 13 phase segments at random points and exchanged the resulting two phase vectors along 5000 realizations, thus preserving the original nonstationarities (Canolty et al., 2006). In multisite recordings, gamma modulation indices were evaluated for each layer separately both from LFP and CSD signals. In tetrode recordings, the modulation index from each tetrode was estimated as the mean modulation index calculated from the less multiunit-contaminated wire over different sessions.

### Unit isolation

Single units were isolated from tetrode recordings using cluster-cutting techniques (OFS, Plexon; and Klustakwik version 1.6). The initial discrimination of clusters and manual refinement was performed using principal components, spike amplitude, and other parameters. To verify cluster quality isolation, the probability of cluster overlapping was calculated as described previously (Hill et al., 2011). Spike trains were analyzed by generating interval time histograms and temporal autocorrelograms (±0.025 s). Only units with >100 spikes, none of them in the refractory period of the interspike time histogram (1–2 ms), and with spike amplitudes three to four times above background noise, typically 20–30 µV, were included. Putative pyramidal cells and interneurons were differentiated following our previous criteria (Bellistri et al., 2013), including trough-to-peak duration of the nonfiltered spike (automatic classification threshold, 0.4 ms), an asymmetry index (0.4; Bellistri et al., 2013), and the first moment of the autocorrelograms (autocorrelation peak classification threshold at 10 ms; Csicsvari et al., 1999). The firing rate was not used as a classificatory criterion, given the large variability, especially between interneuronal types and states (Lapray et al., 2012; Katona et al., 2014). The initial automatic classification was refined in some units based on a holistic evaluation of their activity with additional criteria (recording depth as judged from sharp-wave ripples and phase locking to theta; see below). Units that could not be clearly separated were left unclassified.

### Unit phase-locked firing and rhythmicity analysis

We evaluated theta phase-locking behavior of unit firing by using phase histograms of spike timing around theta peaks recorded at SP. Phase histograms were represented as firing probability from −2π to 2π by dividing each phase bin (8.7º bin size) by the total number of spikes. Phase locking was quantified using the mean vector length of phase distribution, which is a measure of phase consistency between spikes and LFP from 0 to 1. A minimum of 1000 spikes was required to estimate this measure accurately.

To evaluate theta rhythmicity of unit firing, we estimated the power spectrum of the autocorrelogram built at ±0.5 s windows (1 ms bin size). A theta autocorrelation index was defined from the normalized area in the 4–12 Hz band. We also evaluated gamma rhythmicity similarly, by computing the power spectrum of autocorrelograms in the 40–90 Hz band to define a gamma autocorrelation index.

### Simulation of theta phase-locked firing

We ran simulations of theta phase-modulated firing to evaluate the sensitivity of indices of theta phase locking (i.e., the phase vector length and autocorrelation index). Spikes (2000–2500 spikes in a 300 s window) with different degrees of phase locking to a sinusoidal theta wave (8–9 Hz) were used. We evaluated the effect of mean firing rate reduction in the synthetic time series by randomly deleting a fixed proportion of individual spikes every run (100 simulation runs). The large number of spikes (>1500) used ensures a poor effect of spike counts on measures. Phase and autocorrelogram distribution histograms similar to those used for unit data were computed for each simulation run. The mean ± 95% confidence interval value of the phase vector length and the autocorrelation index were reported.

### Interneuronal type classification

We exploited cell type-specific features of firing dynamics that were previously described for neurochemically identified hippocampal GABAergic interneurons to subclassify putative interneurons from tetrode recordings (Somogyi and Klausberger, 2005). Theta-phase preference and firing during SPW ripples were used as classificatory criteria (Czurkó et al., 2011; Diba et al., 2014). The preferred theta phase of each unit was transformed into four categories according to whether the unit fired maximally (mean ± SD) at the peak (±0.43 radians, 25º), at the trough (from ±1.8 to ±3.14 radians, 103–180º), at the descending phase (0.61–1.8 radians, 35–103º included), or at the ascending phase (0.61–1.8 radians, 35–103º included) of theta cycles recorded at SP. Firing during SPW ripples were quantified as the ratio between the basal firing rate (from 200 to 100 ms before the SPW event) and the mean firing rate around the ripple peak (±50 ms) using 1-ms-bin per event smoothed (boxcar 3 points) histograms. SPW ripple events (>10/unit) were identified in tetrode recordings using standard semiautomatic approaches applied to filtered signals (100–600 Hz), as previously reported (Valero et al., 2015). The significance of the SPW/ripple ratio was tested against 1 for each unit using a one-sample *t* test (*p* < 0.05). We also exploited the behavior-dependent specialization of identified interneurons to subclassify them according to their firing dynamics during run/stop transitions (Lapray et al., 2012; Katona et al., 2014). To this purpose, we identify transitions between running and immobility periods using the tracking signal (>10) and estimated a run/stop ratio by calculating the mean firing rate around the transition period (±0.5 s) from 5 ms bin histograms smoothed with boxcar averaging (3 points). Significance of the run/stop ratio was tested against 1 for each unit using a one-sample *t* test similar, as before.

Putative GABAergic interneurons were subclassified as PV and cholecystokinin (CCK) basket cells, and oriens lacunosum moleculare (OLM) and bistratified interneurons based on the following criteria: PV basket cells fired in the descending phase of theta and had SPW/ripple ratios that were significantly >1 and run/stop ratios were significantly >1 (Somogyi and Klausberger 2005; Lapray et al., 2012); CCK basket cells fired at the theta peak and did not consistently display SPW ripples (one-sample *t* test not significant; Somogyi and Klausberger, 2005), and their behavior at run/stop transitions is not reported, and no criterion was adopted; OLM interneurons fired at the theta trough, had SPW/ripple ratios significantly <1, and did not significantly modulate firing during run/stop transitions (run/stop ratios were not significantly different from 1; Somogyi and Klausberger, 2005; Katona et al., 2014); and bistratified interneurons fired at the trough of theta, and had SPW/ripple ratios significantly >1 and run/stop ratios <1 (Somogyi and Klausberger 2005; Katona et al., 2014).

Interneurons that did not meet these criteria were not subclassified. Classification was performed manually based on the quantitative criteria described above.

To validate some of these classification criteria in the TLE context, we obtained intracellular recordings in urethane-anesthetized normal and epileptic rats (1.2 g/kg), as previously reported (Valero et al., 2015). Briefly, anesthetized rats were fastened to a stereotaxic frame and body temperature was kept constant at 37º. A 16-channel silicon probe (100 µm resolution; NeuroNexus) was used to record LFPs from different hippocampal layers. Cells were targeted at the dorsal CA1 with sharp pipettes (1.5 mm/0.86 mm outer/inner diameter, borosilicate glass; A-M Systems) filled with 1.5 m potassium acetate and 2% Neurobiotin (Vector Laboratories) for subsequent histological verification. Recorded interneurons (as judged by their electrophysiological features) were successfully recovered for histological studies, including immunostaining analysis in five control rats (untreated) and four epileptic rats.

### Tissue processing and immunohistochemistry

After completing recordings, rats were transcardially perfused with 4% paraformaldehyde and 15% saturated picric acid in 0.1 m, pH 7.4 PBS. Brains were postfixed overnight at room temperature (RT), washed in PBS, and serially cut in 70 µm coronal sections (VT 1000S Vibratome, Leica). Sections containing the probe and tetrode tracks were identified with a stereomicroscope (S8APO, Leica). Sections containing Neurobiotin-labeled cells were localized by incubating them in 1:400 Alexa Fluor 488-conjugated streptavidin (catalog #016-540-084, Jackson ImmunoResearch) with 0.5% Triton X-100 in PBS (PBS-Tx) for 2 h. Sections were analyzed using an inverted epifluorescence microscope (DMI6000B, Leica). Those containing the recorded soma were selected for immunofluorescence characterization.

For immunohistochemistry, we used a battery of antibodies including monoclonal anti-NeuN (1:1000; catalog #MAB377, BACHEM), polyclonal anti-netrinG1 (1:10) and anti-netrinG2 (1:100; both gifts from S.Itohara from RIKEN, Tokyo, Japan), polyclonal rabbit anti-PCP4 (1:300; catalog #HPA005792, Sigma-Aldrich), monoclonal mouse anti-PV (1:4000; catalog #PV-235, Swant), and polyclonal rabbit anti-somatostatin-14 (1:2000; catalog #T4103, Peninsula). Some sections were also stained with bisbenzimide or a Nissl reaction with toluidine blue. Immunostaining was performed on floating sections using either the biotin–avidin–peroxidase method or fluorescent secondary antibodies.

For silicon probe recordings, NeuN and netrins G1 and G2 were used to identify electrode tracks across layers and regions (Laurent et al., 2015). For tetrodes, we used NeuN for similar purposes and PCP4 to delineate the limits between CA1 and CA2 regions (Valero et al., 2015). The approximate coordinates of all electrode tracks were inferred from the Paxinos atlas using typical anatomical landmarks. We assigned each electrode track as proximal (close to CA2) or distal (close to the subiculum) in the CA1 region, as previously described (Laurent et al., 2015; Fig. 2*D*). In tetrode recordings, tracks were followed along the dorsoventral axis to identify the deepest targeted region. Tracks that did not penetrate the hippocampus were discarded, independent on whether theta phase-locked units were detected.

The identity of intracellularly recorded cells was confirmed by colocalization of different neurochemical markers and streptavidin. Sections containing the somata of the recorded cell were immunostained against PV and somatostatin overnight followed by incubation for 2 h at RT with appropriate secondary antibodies including goat anti-rabbit Alexa Fluor 633 (1:500; catalog #A21070, Molecular Probes), and goat anti-mouse Rhodamine Red (1:200; catalog #115-295-003, Jackson ImmunoResearch) in PBS-Tx and 1% FBS. Streptavidin-positive recorded cells were identified using a confocal microscope (SP5, Leica; with Lens Application System AF software version 2.6.0 build 7266, Leica Microsystems), as previously described (Valero et al., 2015).

### Statistical analysis

Statistical analysis was performed either with the SPSS software (IBM) or with MATLAB. No statistical methods were used to predetermine sample sizes, but they were similar to those reported previously (Inostroza et al., 2013; Laurent et al., 2015).

Normality was confirmed for spectral indices (either directly or after logarithmic transformation) using the Kolmogorov–Smirnov test. Homoscedasticity was tested using the Levene’s test. Gamma power values from each band (either detrended or in absolute values) were analyzed with ANOVAs of various factors (group, gamma band, and recording location along the proximodistal axis). To quantify theta–gamma modulation power measurements estimated from LFP and CSD, signals from each gamma band were compared among groups, layers, and location for a given band, and between bands and groups from different layers and phases. Theta phases were restricted to the four central phases around the peak recorded at SP (the central one, the one before, and the two after), to include only one theta cycle in the analysis. Comparisons between groups and proximodistal location were evaluated using Welch’s *t* tests. Correlation between electrophysiological indices or spectral and behavioral indices was evaluated with the Pearson product-moment correlation coefficient, which was tested against 0 (i.e., no correlation was the null hypothesis) at *p* < 0.05 (two sided). Both the Pearson coefficient and *p* value are reported to facilitate interpretation.

Normality and homoscedasticity (Levene’s test) were not confirmed for firing rate and theta-phase preference data of units sorted from tetrodes. They were analyzed with the Bartlett’s test for variance, and posteriorly compared with Welch’s *t* tests. Rayleigh circular statistics, equivalent to the *t* test, was used to characterize phase-locking behavior whenever required. The phase-locking vector length and theta and gamma autocorrelation indices from units were analyzed with ANOVAs for groups and recording locations, after confirming normality.

Results are represented as the mean ± SD for indices meeting the normality test. Results are represented as whisker-box plots or as mean ± SD values with individual points when normality distribution was not confirmed.

## Results

### Contrasting wide-band differences of CA1 gamma oscillations

To examine layer-specific theta–gamma coupling, we used 16-channel silicon probes to record LFPs from rats engaged in object recognition tasks that tested for the spatial and temporal aspects of episodic-like memory (Materials and Methods; Inostroza et al., 2013). To meet the methodological constraints required for the estimation of CSD signals (Materials and Methods), we used data from either the sample or the test sessions, as we found similar LFP spectral indices (*r* > 0.7, *p* < 0.01 for between-session comparisons; Inostroza et al., 2013). Seizures and epileptiform activities were not recorded during these sessions. A total of 10 normal rats and 8 epileptic rats met these requirements.

We first noted that the typical 1/*f^n^* trend of the LFP power spectrum complicated examination of the full range of gamma activity from 30 to 150 Hz in different layers (Fig. 1*A*; only data from SLM are shown). Different frequency bands did not follow similar scale laws, in contrast to the case when no biological activity was recorded (dead condition; Fig. 1*A*). The *n*-factor that better fitted the 1/*f* power law was band dependent at each layer (Fig. 1*B*, data from SLM; one-way ANOVA for four bands; control rats: *F*_(3,36)_ = 52, *p* < 0.0001; epileptic rats: *F*_(3,28)_ = 61, *p* < 0.0001), possibly reflecting that distinct gamma generators contribute unevenly to scale-free dynamics across different layers (He, 2014). More importantly, differences between groups were found for the *n*-factor that better fitted the 150–500 Hz band (Fig. 1*B*; Student’s *t* test, *p* = 0.0245). Overall, the absolute power poorly accounted for group differences across gamma bands (Fig. 1*C*), as we have reported before (Inostroza et al., 2013).

To overcome these limitations, we estimated the *n*-factor between 30 and 210 Hz to detrend power spectra accordingly at different layers. Removing the linear trend facilitated identification of spectral differences between groups along bands (Fig. 1*D* for SLM), including the typical gamma bands that were previously identified: the gamma-s (30–60 Hz), gamma-m (60–90 Hz), and gamma-f (90–140 Hz; Schomburg et al., 2014). We therefore adopted this approach to quantify gamma oscillations between groups.

### Layer-specific gamma activity distribution in normal and epileptic rats

Theta–gamma dynamics in TLE rats (Fig. 2*A*) were similar to those described in normal animals (data not shown; Scheffer-Teixeira et al., 2012; Schomburg et al., 2014). Short runs of slow gamma waves (30–60 Hz) were typically seen during the descending theta phase at SP (Fig. 2*A*, black arrowheads), while runs of 60–90 Hz (mid-gamma waves) tended to occur at the theta peak (Fig. 2*A*, gray arrowheads). Importantly, the contribution of slow and mid-gamma waves varied dynamically on a cycle-by-cycle basis, possibly reflecting independent activity at different input streams. Given similar phase distribution of theta cycles between groups (Fig. 2*B*), we aimed to look for differences of theta–gamma modulation in TLE using detrended power spectra.

Examination of the distribution of LFP gamma activity across layers showed a typical laminar profile both in control and epileptic rats (Fig. 2*C*). Broadband gamma dominated at hilar regions, while different contributions from slow to fast gamma were found at SR and SLM. Given the strong proximodistal organization of theta and gamma bands in the hippocampus (Schomburg et al., 2014; Laurent et al., 2015), we chose to evaluate quantitatively the contribution of different gamma bands across proximal and distal CA1 layers (Fig. 2*D*; proximal: *n* = 5 control rats, *n* = 4 epileptic rats; distal: *n* = 5 control rats, *n* = 4 epileptic rats).

For LFP signals, we found an effect for group only at the fast gamma band in SP (two-way ANOVA for groups and location, *F*_(1,11)_ = 25.1; *p* = 0.0004; in three rats, the SP was not sampled) and a not significant trend in SR (*F*_(1,14)_ = 4.1; *p* = 0.063; Fig. 2*E*; no proximodistal effect, no interaction). *Post hoc* Bonferroni-corrected Welch’s *t* tests suggested increased fast gamma activity in epileptic rats both at proximal and distal locations (Fig. 2*E*). We also detected group and proximodistal effects on mid-gamma at SLM (group effect: *F*_(1,14)_ = 5.9, *p* = 0.0293; proximodistal effect: *F*_(1,14)_ = 15.5, *p* = 0.0015; interaction, *p* = 0.0067), but these were associated with a decreased gamma power at proximal locations instead (Fig. 2*E*). Importantly, only this decreased mid-gamma band survived in CSD signals of control and epileptic rats (*F*_(1,14)_ = 4.8, *p* = 0.0451 for groups; no proximodistal effects but significant interaction at *p* = 0.0013), suggesting that increased fast gamma activity at SP may be contaminated by neuronal firing (Ray and Maunsell, 2011). No speed or exploratory differences accounted for these effects. Thus, given potential differences of 1/*f* between groups for >150 Hz and discrimination issues affecting the separation of the gamma band from the firing spectral leakage, we chose to adopt techniques that better distinguish the contribution of different gamma CSD generators across theta phases (Schomburg et al., 2014).

### Layer-specific disruption of proximal CA1 slow gamma in TLE rats

**Figure 3.**
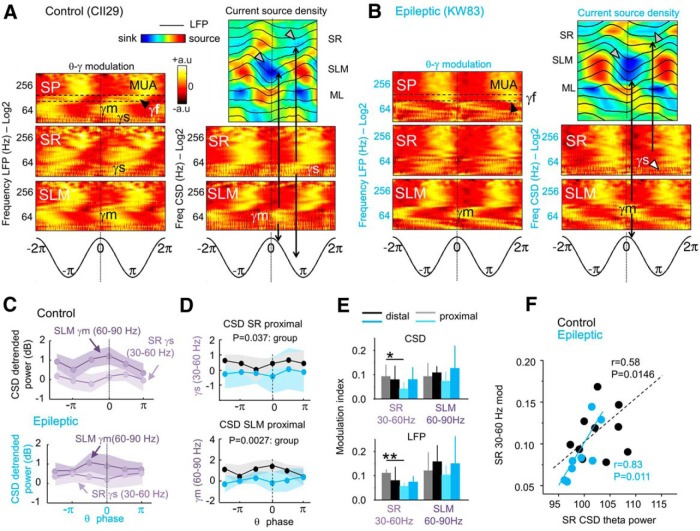
Theta-phase modulation of gamma activity. ***A***, Representative example of theta–gamma modulation recorded from the control rat shown in Figure 2*C*. Theta-phase modulation of LFP gamma activity is shown at left for SP, SR, and SLM channels (black-yellow plots). CSD signals help to account for local gamma generators (blue-red plot at right). Theta-phase modulation of CSD gamma activity is shown below the CSD plot for SR and SLM channels. All signals are aligned by the theta peak recorded at SP (vertical discontinuous line). Note the clear theta-phase segregation of the slow and mid-gamma bands from MUA recorded at the SP. Gamma-s (γs) CSD activity (30–60 Hz) is better isolated at the SR during the descending phase of theta cycles in association with the corresponding SR theta sink (vertical arrows). Gamma-m (γm) CSD activity (60-90 Hz) is isolated at the SLM during the SP theta peak, in association with the corresponding SLM sink. SR and SLM sinks are indicated by gray arrowheads in the CSD plot. ***B***, Same as in ***A*** for the epileptic rat shown in Figure 2*C*. Note the poor slow activity compared with the control rat. ***C***, Phase difference between the SR and SLM CSD signals in all control (top plot, *n* = 10) and epileptic rats (bottom plot; *n* = 8). Note the poor modulation of SR slow gamma in epileptic rats (only data from six phases are shown). ***D***, Group differences of the SR slow and SLM mid-gamma CSD generators were found only at proximal locations. The *p* values refer to group effects in a two-way ANOVA for groups and four phases. ***E***, Differences of the gamma modulation index between groups were found only in the slow 30–60 Hz band at proximal locations both for LFP and CSD signals. *p < 0.05; **p < 0.005. ***F***, Correlation between the LFP slow gamma modulation index at SR and the theta power of the SR CSD signal for both groups together (black) and within the epileptic group (blue).

To dissect the contribution of different gamma generators from high-frequency spectral leakage, we exploited their theta phase and layer specificity validated before with source separation techniques (Schomburg et al., 2014). Different gamma bands segregated across layers and theta phases referenced against SP. In control rats, the power of the slow gamma band was higher during the falling phase, especially at SR for both LFP and CSD signals (Fig. 3*A*). Instead, the mid-gamma band was stronger at SLM during the theta peak. CSD signals at SR and SLM clearly separated these two gamma generators consistently with Schaffer- and temporoammonic-associated sinks observed in CSD plots (Fig. 3*A*, arrows and gray arrowheads). As previously reported (Schomburg et al., 2014), these two gamma bands can be phase segregated in LFP recordings at SP (Fig. 3*A*, top left SP channel), which facilitate their identification with tetrodes. The phase of fast gamma activity (90–140 Hz) was tightly associated with multiunit activity (MUA), making their examination difficult (Fig. 3*A*, black arrowhead at SP).

In epileptic rats, we found approximately similar phase segregation of mid-gamma bands at SLM both in LFP and CSD signals (Fig. 3*B*). However, in contrast to control rats, the slow gamma band was attenuated at the SR (Fig. 3*B*, white arrowhead), which is consistent with smaller Schaffer-associated CSD sinks at the SR of epileptic rats (*p* = 0.0467; Fig. 3*B*, gray arrowheads). The fast gamma band could not be reliably isolated from MUA (Fig. 3*B*, black arrowhead at SP), further supporting the idea that the increased power in TLE shown before reflected contaminated firing (Fig. 2*E*).

We confirmed adequate separation of the slow gamma and at SR and the mid-gamma band at SLM in CSD signals within the control group (within-group, two-way ANOVA for two bands and four phases: *F*_(1,72)_ = 30.5, *p* < 0.0001; Fig. 3*C*, top plot) and the epileptic group (*F*_(1,56)_ = 8.4, *p* = 0.0053; Fig. 3*C*, bottom plot; no phase effect, no interaction). However, the gamma band appeared strongly attenuated in TLE rats. This was further supported by comparing each laminar gamma generator at proximal and distal locations separately, using a two-way ANOVA for groups and four phases. We found that statistical differences concentrated only at proximal layers (Fig. 3*D*). The power of both the slow gamma band (*F*_(1,28)_ = 4.8, *p* = 0.0373; Fig. 3*D*, top plot) and the mid-gamma band (*F*_(1,28)_ = 17.3, *p* = 0.0027; Fig. 3*D*, bottom plot) was reduced in TLE rats at the proximal SR and SLM, respectively. An index of theta-phase gamma modulation was computed for the slow and the mid-gamma bands in CSD and was statistically tested against 5000 surrogates computed by random-phase transposition to preserve unspecific nonstationarities of the data (see Materials and Methods). Theta phase-amplitude modulation of slow and mid-gamma band activity was confirmed statistically both in control rats (*p* < 0.00001) and epileptic rats (*p* < 0.001). Importantly, we detected group differences of modulation indices at proximal locations for the slow gamma band (*F*_(1,14)_ = 3.7, *p* = 0.0445; two-way ANOVA for groups and location) but not for mid-gamma band (Fig. 3*E*, top plot) CSD, with LFP signals demonstrating similar effects (*F*_(1,14)_ = 5.7; *p* = 0.012; Fig. 3*E*, bottom plot). The LFP modulation index of the slow gamma band at SR significantly correlated with the theta power of CSD signals measured at this layer in TLE rats (*r* = 0.83, *p* = 0.011) and in both groups (*r* = 0.58, *p* = 0.0146; Fig. 3*F*), suggesting that its disruption may reflect weaker activation of CA1 by Schaffer collaterals.

Therefore, using phase-decomposition methods we identified a specific discoordination between theta and slow gamma (30–60 Hz) oscillations at the proximal SR in TLE rats. Theta-phase preference of CA1 gamma band has been associated with specific mechanisms of CA3 and entorhinal cortical inputs during the encoding and retrieval of information (Schomburg et al., 2014; Trimper et al., 2014; Zheng et al., 2016). We thus asked whether their disruption in TLE may be associated with episodic-like memory dysfunction reported in this condition (Inostroza et al., 2013; Lega et al., 2015).

### Disruption of theta–slow-gamma coupling linked to memory deficits

**Figure 4.**
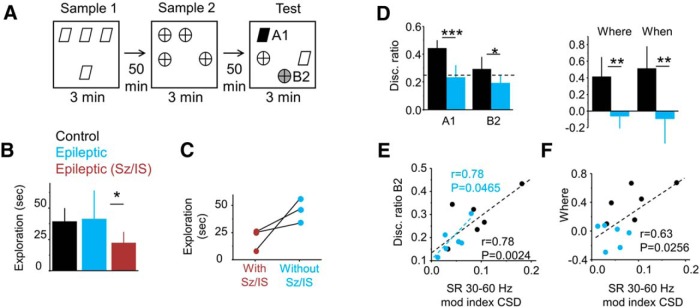
Relationship between slow gamma (30–60 Hz) and performance in the what-where-when episodic-like memory task. ***A***, Schematic representation of the task. The scheme shows object configurations in the sample and test phases (3 min each). The retention interval is 50 min. ***B***, Group data (mean ± SD) of the total exploratory time during the test phase for control rats (*n* = 6), epileptic rats tested in periods free of epileptiform activities (*n* = 6), and epileptic rats exhibiting seizures and/or interictal spikes within 2 h of the behavioral task (*n* = 4; epileptic Sz/IS group), as judged from simultaneous multisite silicon probe recordings. Note the reduced exploratory behavior of rats experiencing epileptiform activities compared with their Sz/IS free epileptic mates. *p < 0.05. ***C***, Data from three rats evaluated in the what-where-when task during periods with and without epileptiform events. Note the different exploratory behavior in the very same animals performing the task with or without signs of epileptiform activities in the dorsal hippocampus. ***D***, Group data (mean ± SD) for the discrimination ratio of object A1 (black) and B2 (green in ***A***), carrying most of the temporal and spatial memory of the exploratory episode. Note epileptic rats discriminate at chance level (0.25). Right plot shows group data for the spatial and temporal memory indices, where and when, respectively. *p < 0.05; **p < 0.005; ***p < 0.0001. Data from six control rats and six epileptic rats free from epileptiform activities. ***E***, Correlation between the slow gamma CSD modulation index and the discrimination index for object B2 (***A***, green). Significant Pearson correlation was found for both groups (black) and the epileptic group alone (blue). ***F***, Correlation between the slow gamma CSD modulation index and a spatial memory index (where) for both groups (black), but not for either group alone.

Cognitive associates of theta–gamma disruption were investigated by examining the correlation between spectral and cognitive indices derived from the episodic-like memory task (what-where-when) in six control rats and six epileptic rats (the remaining four control rats and two epileptic rats performed a different version of recognition tasks, see Materials and Methods). During these tasks, rats discriminate between objects carrying different degrees of novelty (Fig. 4*A*), requiring spatial and temporal recognition memory abilities (Kart-Teke et al., 2006). Importantly, these animals were free of seizures and interictal activities during the entire session.

First, we aimed to quantify the effect of epileptiform activities in cognitive performance. Epileptic animals free of epileptiform events within 2 h of the task (*n* = 6) explored similarly to control rats (*n* = 6; Fig. 4*B*, black vs blue). In contrast, when seizures and/or interictal activities were recorded (*n* = 4 epileptic rats), animals tended to be less exploratory compared with their epileptic mates (*p* = 0.0087; Fig. 4*B*, red vs blue). Three of these rats were re-evaluated in the task on different days, allowing for a longitudinal analysis when no seizures or interictal activities were recorded. They exhibited improved exploration (*p* = 0.0157, paired *t* test; Fig. 4*C*). Thus, we concluded that with our experimental approach we were able to weight LFP oscillations over peri-ictal effects on cognitive performance.

As previously reported for another group of animals (Inostroza et al., 2013), epileptic rats free of any sign of epileptiform events, explored objects A1 and B2 of the what-where-when task at chance levels in contrast to normal rats (Fig. 4*D*, left plot). Group statistical differences concentrated in the discrimination index of A1 (*p* < 0.0001) and B2 (*p* = 0.0293). The other two objects (Fig. 4*A*) were not discriminated differently between groups (data not shown). Differences were also detected for the where (*p* = 0.0013) and when indices (*p* = 0.0024; Materials and Methods) of the spatial and temporal components of recognition memory (Fig. 4*D*, right plot), as previously reported (Inostroza et al., 2013).

Individual variability in the TLE group and between-group difference of animal performance could be explained by variability in the slow gamma modulation index at SR from CSD signals (Fig. 4*E*). We found significant Pearson correlation between this modulation index and discrimination abilities for B2 within the epileptic group (*r* = 0.78, *p* = 0.0465) and for both groups together (*r* = 0.78, *p* = 0.0024; Fig. 4*E*). We also found a strong effect for both groups in the where index but not for either group alone, preventing us from forming a conclusion due to a clustering effect that dominated the correlation (Fig. 4*F*). No correlation was found between the slow gamma modulation index and any index for object A1.

Thus, disruption of CA1 theta–slow-gamma coupling at the proximal SR linked to spatial memory deficits in TLE, reflecting a microcircuit rhythmopathy. Recent data suggest that input pathways and interneuron–pyramidal cell interactions determine the organization of theta-nested slow gamma oscillations (Mizuseki et al., 2009; Lasztóczi and Klausberger, 2014; Schomburg et al., 2014). We thus looked for the mechanisms of theta–gamma disruption in TLE at the single-cell level.

### Disrupted firing rate in the epileptic hippocampus

**Figure 5.**
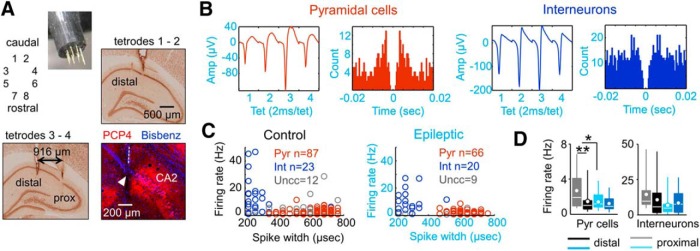
Tetrode recordings of single cell activity. ***A***, Eight independent tetrodes were advanced into the dorsal hippocampus to target CA1 cells at the stratum pyramidale of normal and epileptic rats. Sections immunostained against NeuN were used to identify proximal and distal recording locations. Immunostaining against the CA2-specific protein PCP4 was used to validate proximal CA1 locations. Blue is bisbenzimide used to stain cell nuclei. ***B***, Typical spike waveform and autocorrelogram of a putative pyramidal cell (red) and an interneuron (blue) recorded from an epileptic rat (KWGAT6). ***C***, Units were classified according to several criteria, including spike width data. A group of units remained unclassified (gray). The number of units is given in the figure. Data from three control and three epileptic rats. ***D***, Firing rate data from putative pyramidal cells and interneurons recorded at proximal and distal locations in control and epileptic rats. *p < 0.05; **p < 0.001.

Tetrodes were used to isolate firing from PCs and interneurons from the proximal and distal CA1 during object recognition and pellet-chasing tasks (Fig. 5*A*,*B*; *n* = 3 control rats; *n* = 3 epileptic rats). We sorted 87 PCs (45 proximal PCs, 42 distal PCs) and 23 interneurons (13 proximal interneurons, 10 distal interneurons) from control animals. A total of 66 PCs (35 proximal PCs, 31 distal PCs) and 20 interneurons (10 proximal interneurons, 10 distal interneurons) were sorted from epileptic rats. Some units were left unclassified (*n* = 12 control rats, *n* = 9 epileptic rats) and were not included in the analysis (Fig. 5*C*).

PCs from normal rats exhibited proximodistal differences in the mean firing rate, with lower values at the distal CA1 (Welch’s *t* test, *p* = 0.0021), similar to recent reports (Oliva et al., 2016). In contrast, such a proximodistal segregation of PC firing rate was not present in epileptic rats (Fig. 5*D*, left; *p* = 0.6189). A Bartlett’s test for variance across groups and locations suggested a strong difference in the variability of firing rate values of PCs (×2(3) = 17.9, *p* = 0.0005). Despite this variability, we found a lower mean firing rate in proximal PCs of the epileptic group compared with those in the control group (Fig. 5*D*, left; Welch’s *t* test, *p* = 0.0045). No group (*p* = 0.1340) or proximodistal effects (*p* = 0.2898) were confirmed for putative interneurons, which exhibited large variability within groups (Fig. 5*D*, right).

### Reduced theta modulation of CA1 proximal interneurons in TLE rats

**Figure 6.**
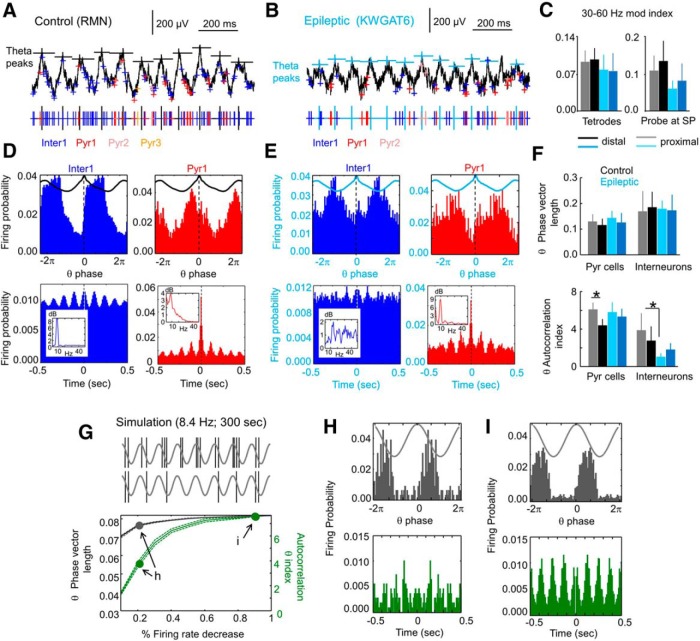
Disruption of interneuronal firing patterns at the proximal CA1 of epileptic rats. ***A***, Representative tetrode recording from a control rat (only one channel is shown). Spikes from individually sorted pyramidal cells and one interneuron are shown in different colors. Large black ticks mark the position of theta peaks at SP. ***B***, Same as in ***A*** for an epileptic rat. ***C***, Proximodistal group differences (mean ± SD) of slow gamma modulation index as obtained from tetrodes (eight proximal and eight distal from three control rats, and six proximal and six distal from three epileptic rats) and silicon probes (four proximal and five distal from nine control rats; and three proximal and three distal from six epileptic rats). Different degree of spike contamination in tetrode and silicon probe recordings likely prevents a quantitative comparison between these datasets. ***D***, Theta phase-locked firing (top row) of the interneuron and pyramidal cell shown in ***A***. Firing phase modulation (8.7º bin size) was quantified using the theta-phase vector length. Bottom histograms represent the unit firing autocorrelograms (1 ms bin size). Insets show the power spectrum of the unit autocorrelogram, from which a theta autocorrelation index was estimated. This interneuron was subclassified as a putative PV basket cell (Table 1, unit 0405RMN_OF_T1R-01_part1_1). ***E***, Same as in ***D*** for the interneuron and pyramidal cell shown in the epileptic rat in ***B***. The putative interneuron could not be quantitatively subclassified, but it resembled an OLM interneuron (Table 1, unit 2911KWGAT6_OF_1_btp_01_T5R15-03_3). ***F***, Group data (mean ± SD) for the theta-phase vector length (top plot) and the theta autocorrelation index (bottom) for all units. Note differences between groups concentrated at the autocorrelation index for proximal interneurons. *p < 0.05. ***G***, Computer simulation of theta phase-locked firing with different degrees of firing rate (mean ± SD from 100 simulations). Decreasing the global firing rate had a stronger impact in the autocorrelation index than in the phase vector length, indicating different sensitivity of these measures. Arrows point to simulations shown at left. ***H***, ***I***, Data from simulations indicated by arrows in ***G***. Both the theta-phase histograms (top row) and the autocorrelograms (bottom) are shown. Note poor rhythmicity in the autocorrelogram but not apparent change in theta-phase firing preference when the global firing rate decreases to <20%.

We next asked about theta phase-locking behavior of individual PCs and interneurons in control rats (Fig. 6*A*) and epileptic rats (Fig. 6*B*). To analyze spike–field interactions, we chose units with >1000 spikes in the recording session (>300 s) and with at least one tetrode wire suitable for spectral analysis (57 control PCs: 24 proximal PCs, 33 distal PCs; 37 epileptic PCs: 12 proximal PCs, 25 distal PCs; 19 control interneurons: 10 proximal interneurons, 9 distal interneurons; 16 epileptic interneurons: 8 proximal interneurons, 8 distal interneurons). First, we confirmed similar trends in the slow gamma modulation index in LFP signals in rats recorded with tetrodes versus those recorded with multisite probes (Fig. 6*C*). Differences between tetrode and silicon probe data were likely to be related to technical issues affecting these recording modalities, preventing quantitative comparisons.

We noted different phase-locking behavior, especially for interneurons in epileptic rats (Fig. 6, compare *D*, *E*). The phase vector length failed to capture differences between groups and exhibited large variability (Fig. 6*F*, top plots). Instead, the autocorrelation index appeared more sensitive to group differences for interneurons (two-way ANOVA for groups and locations: *F*_(1,31)_ = 6.8, *p* = 0.0163; Fig. 6*F*, bottom plot; no proximodistal effects, no interaction). For PCs, proximodistal effects (*F*_(1,90)_ = 5.9, *p* = 0.0168), but not group effects, dominated statistical differences (no interaction).

To better understand this point, we investigated the sensitivity of the two theta modulation indices to capture changes in firing behavior using synthetic spikes (100 random simulations; Materials and Methods). We chose to evaluate the effect of reducing the mean firing rate to simulate trends detected in control and epileptic rats (Fig. 5*D*). The phase vector length was less sensitive to decreases in the mean firing rate than the autocorrelation index (Fig. 6*G*, independent on the spike count), especially for cells exhibiting initially strong theta phase-locked firing. A decrease in the basal firing rate had a stronger impact on the autocorrelation index than a decrease in the mean phase vector length (Fig. 6*H*,*I*).

Thus, our data suggest that theta-modulated firing of proximal CA1 interneurons is reduced in the epileptic hippocampus, pointing to specific mechanisms. However, different sensitivities of modulation indices and individual variability between interneuronal types precluded strong group differences. Given the role of proximal PV basket cells in slow gamma oscillations at the falling theta phase (Lasztóczi and Klausberger, 2014), we asked for more specific links with decreased slow gamma modulation in TLE.

### Specific alteration of theta and gamma rhythmicity of PV basket cells

PV-positive interneurons represent a major population involved in the generation of gamma oscillations (Wulff et al., 2009; Zemankovics et al., 2013). Importantly, recent data suggest that PV basket cells in particular are critical for theta-nested slow gamma bands at the proximal CA1 (Lasztóczi and Klausberger, 2014). CA1 PV basket cells have their somata between the stratum oriens and the cell body layer, and therefore are easily accessible with tetrodes. Considering their phase-firing preference and the specific disruption of slow gamma oscillations at the falling phase of theta in TLE rats, it was tempting to examine the specific behavior of this interneuronal population more closely.

To evaluate this point further, we exploited cell type-specific firing features and subclassified putative interneurons recorded with tetrodes in four different classes (i.e., PV or CCK basket cells, OLM and bistratified interneurons; Materials and Methods). This approach was adopted previously (Czurkó et al., 2011; Diba et al., 2014), but, instead of relying only on theta and SPW ripples, we also analyzed behavioral dependency during runs and stops (Lapray et al., 2012; Katona et al., 2014).

**Figure 7.**
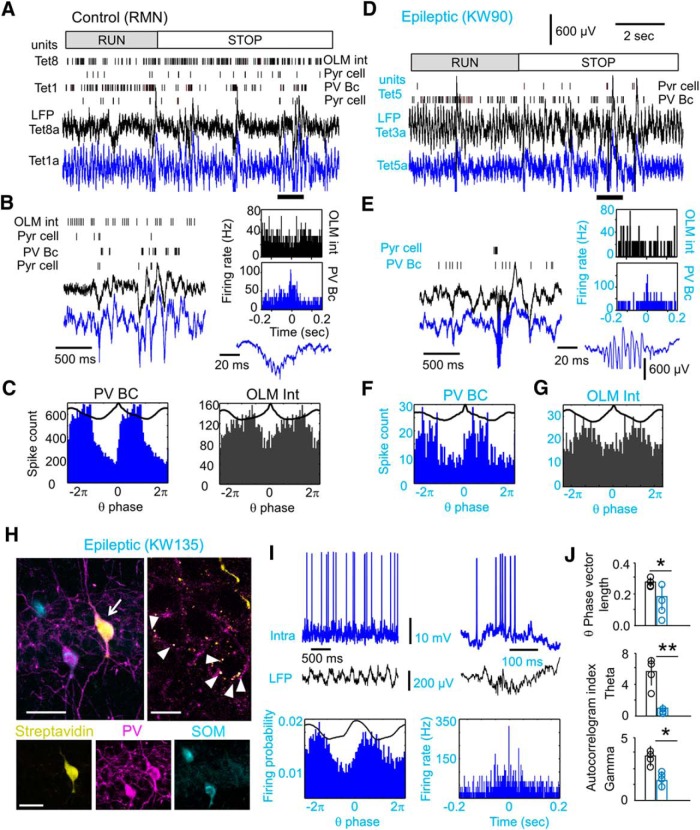
Rhythmic firing of proximal parvalbumin basket cells is specifically disrupted in epileptic rats. ***A***, Putative interneurons were subclassified according to criteria previously confirmed in identified types, including PV basket cells (PV-BC) and OLM interneurons. Firing dynamics at run/stop transitions was one of these criteria. An example is shown for two tetrodes from a control rat. Note poor firing modulation at run/stop transition of the interneuron in tetrode 8 (OLM; Table 1, unit 0505RMN_TrD_1_T8_1) vs strong modulation of the interneuron in tetrode 1 (PV-BC; unit 0505RMN_TrD_1_T1-03_1). The second unit shown at each tetrode was a putative pyramidal cell. Bottom traces show LFP from one channel from each tetrode. ***B***, Firing of the units shown before during SPW ripples enlarged from ***A*** (thick line). Note different participation of interneurons classified as OLM and PV-BC in the perievent histograms at right. One of the ripple events is expanded at the bottom. ***C***, Theta phase-locked firing of the units shown before. ***D***, Same as in ***A*** for a putative PV basket cell in an epileptic rat showing modulation by run/stop transition (Table 1, unit 1612KAW90_OF_T5R16-01.mat_2). ***E***, Same as in ***B***. Histograms show SPW-triggered firing of the PV-BC shown at left and that of the putative OLM cell shown in ***G***. Note that large-amplitude, high-frequency oscillations typical of the epileptic hippocampus that saturate the voltage scale shown here. ***F***, Theta-phase firing histogram of the putative PV-BC shown before. ***G***, Theta-phase firing histogram of a putative OLM interneuron from rat KWGAT6 (unit 2911KWGAT6_OF_1_btp-01_T4-01_1). ***H***, Neurochemically identified PV basket cell recorded intracellularly from an epileptic rat (arrow; scale bar, 50 µm). Note PV-positive synaptic boutons around pyramidal cell somata shown at right (arrowheads, two 2-µm-thick optical section; scale bar, 15 µm) and negative immunoreactivity to somatostatin (SOM) at bottom (scale bar, 50 µm). ***I***, Activity of the PV basket cell shown in ***H*** during theta (left) and SPW ripples (right) validate classification criteria in the context of TLE. ***J***, Theta-phase vector length and autocorrelation index for theta and gamma from all proximal putative PV basket cells (*n* = 4 control rats, *n* = 4 epileptic rats) confirm preferential disruption of their rhythmicity in TLE. *p < 0.05; **p < 0.001. See also Table 1.

Using these criteria, we could identify 10 of 19 control units and 7 of 16 epileptic units, with firing behavior compatible with either of these classes (Table 1). In normal rats, putative PV basket cells (*n* = 4) and OLM interneurons (*n* = 2) exhibited separate behavior during run/stop transitions (Fig. 7*A*) and SPW ripples (Fig. 7*B*), and were differently segregated in phase during theta cycles (Fig. 7*C*), which is consistent with neurochemically identified cells (Somogyi and Klausberger, 2005; Lapray et al., 2012; Katona et al., 2014). Activity from other units in control rats were more compatible with CCK basket cells (*n* = 2) and bistratified interneurons (*n* = 2), while the remaining putative interneurons could not be unequivocally subclassified (*n* = 9; Table 1).

**Table 1:** Firing characteristic of all extracellularly recorded CA1 interneurons and their putative classification

Unit	Location	Firing rate (Hz)	Theta-phase vector index	Theta autocor index	Gamma autocor index	Theta-phase class	SPW/ripple ratio	Run/stop ratio	Classification
Control									
0405RMN_OF_T1R-01_part1_1	p	45.94	0.30	7.09	2.37	Desc	**2.05**	**0.38**	**PV-BC**
0505RMN_TrD_1_T1-03_1	p	17.06	0.28	3.16	3.74	Desc	**2.21**	**0.37**	**PV-BC**
0505RMN_TrD_1_T8_1	p	5.65	0.10	3.28	0.19	Trough	**0.62**	1.41	OLM
1011RMP_OF_cond1-2_T2R17-02_2	p	6.05	0.10	1.10	1.42	Trough	1.24	1.03	Unclassified
1205RMN_4_btp_T2R1-04_aligned_1	p	16.14	0.06	1.83	1.05	Desc	1.31	**0.87**	Unclassified
1205RMN_4_btp_T7R31-01_1	p	9.58	0.08	2.87	1.94	Desc	1.06	1.34	Unclassified
H_1304RMN_TrD_1_btp_Tets_T7_1	p	10.90	0.09	2.39	1.62	Desc	0.85	1.12	Unclassified
1404RMN_T7R31_1	p	7.39	0.26	7.54	3.57	Desc	**3.08**	**0.19**	**PV-BC**
1412RMP_OFEM_4_T7R_3	p	7.53	0.11	2.69	2.91	Trough	**0.14**	1.34	OLM
1504RMN_TrD_T5-04_1	p	35.18	0.31	5.89	3.89	Peak	1.64	**0.38**	CCK-BC
0405RMN_OF_T4R6-04_spl_t001-01_1	d	15.14	0.26	2.99	1.94	Trough	**3.42**	**0.30**	Bistratified
0505RMN_TrD_1_T4-02_2	d	0.49	0.26	1.89	2.13	Trough	1.67	**0.10**	Unclassified
H_1307RMO_TrD_mrg_sil_T3_1	d	1.92	0.23	4.16	5.71	Trough	**4.11**	**0.72**	Bistratified
H_1307RMO_TrD_mrg_sil_T4_r_1	d	1.65	0.16	5.70	3.36	Asc	**4.95**	**0.63**	Unclassified
1404RMN_T4R-01_1	d	44.47	0.24	0.67	4.62	Desc	1.06	1.03	Unclassified
1407RMO_OF_T4-02_1	d	1.65	0.11	0.56	3.33	Trough	1.83	1.19	Unclassified
1711RMP_OF_c12_T3_p1_r_m-01_3	d	5.65	0.11	1.55	1.86	Trough	1.10	1.01	Unclassified
0912RMPTet5Cell3	d	15.43	0.26	5.69	3.86	Desc	**2.01**	**0.74**	**PV-BC**
0912RMP_OFEM_T4-01_1	d	15.30	0.12	0.85	1.69	Peak	2.47	2.86	CCK-BC
Epileptic									
0412KAW90_OF21_T8-01.mat_1	p	2.36	0.17	0.59	1.38	Desc	**1.60**	**0.54**	**PV-BC**
0712KWGAT6FEM_2_btp-01_T5_3	p	1.51	0.20	2.20	1.26	Desc	**2.37**	**0.01**	Unclassified
0912KAW90_OF-01_T5R16-01_1	p	0.44	0.15	1.05	1.24	Trough		3.39	Unclassified
1312KAW90_OF_T5R16-01_1	p	14.69	0.24	0.67	1.80	Desc	**1.79**	**0.32**	**PV-BC**
1612KAW90_OF_T5R16-01.mat_2	p	6.01	0.22	1.19	0.53	Desc	**1.96**	**0.25**	**PV-BC**
2109gfp4_OF_3_T6R7-02_rev_2	p	4.87	0.07	3.22	1.64	Peak	1.21	**0.48**	CCK-BC
2911KWGAT6_OF_1_btp-01_T5R15-03_3	p	7.41	0.15	0.68	5.45	Trough	1.13	0.93	Unclassified (OLM)
2610KWGAT6Tet6Cell1	p	26.42	0.06	1.54	2.27	Desc	**1.54**	**0.51**	**PV-BC**
0712KWGAT6Tet1Cell2	d	26.21	0.21	4.75	0.99	Trough		**0.62**	Unclassified
0610gf4_OF_T8_3	d	0.21	0.15	3.68	1.82	Asc		0.95	Unclassified
0712KWGAT6FEM_2_btp-01_T4_2	d	7.51	0.13	1.26	0.99	Trough	**1.72**	0.31	Unclassified (bist)
H_1810gf4_Trd_1_btp_T8R1-01_2	d	14.61	0.11	4.68	2.99	Trough	**11.71**	**0.14**	Bistratified
2511KWGAT6_OF_1_T8R20-05_rev_2	d	20.02	0.25	2.62	4.95	Trough		**0.71**	Unclassified (PV)
2511KWGAT6_OF_1_T8R20-05_rev_4	d	9.80	0.20	2.09	1.97	Trough		0.41	Unclassified (bist)
2911KWGAT6_OF_1_btp-01_T4-01_1	d	1.60	0.10	1.07	0.95	Trough	**0.38**	0.64	OLM
1812KAW90_OF-01_T2R1-01 (1)	d	2.03	0.30	5.79	3.17	Trough	**2.46**	1.87	Unclassified
Between-group comparisons									
**Control vs epileptic proximal (*p* value)**		0.1620	0.8009	**0.0122**	0.6224		0.3825	0.9131	
Control vs epileptic distal (*p* value)		0.8601	0.6724	0.5505	0.1881		0.3974	0.4663	
Control vs epileptic (*p* value)		0.2416	0.6582	0.1664	0.2048		0.4184	0.5657	
**Control PV-BC vs epileptic PV-BC (*p* value)**		0.3979	**0.0439**	**0.0030**	**0.0115**		0.0624	0.8969	
**Control PV-BC vs epileptic PV-BC proximal (*p* value)**		0.3817	0.0737	**0.0091**	**0.0127**		0.0556	0.4002	

prox, Proximal; dist, distal; asc, ascending; desc, descending; autocor, autocorrelogram; PV-BC, PV-positive basket cell; CCK-BC, CCK-positive basket cell; bist, bistratified. Bold numbers indicates values that are statistically significant at 0.05 for the SPW ripple and run/stop ratios, and for comparisons between groups. Units were subclassified according to criteria given in the text (see Materials and Methods). Units shown in Figure 7 are highlighted at the Classification column.

We found putatively similar PV basket cell behavior in several units recorded from epileptic rats (Fig. 7*D–F*, blue), despite the fact that large-amplitude fast ripples typical of the TLE hippocampus compromised sorting during some events (Fig. 7*E*; Ibarz et al., 2010; Merricks et al., 2015). Actually, the SPW/ripple ratio (Materials and Methods) had a nonsignificant trend to lower values in putative PV basket cells from epileptic rats (Student’s *t* test, *p* = 0.0624), suggesting their weak feedforward activation by Schaffer collaterals. Thus, we validated our classification criteria for PV basket cells in the TLE context with intracellular recordings from urethane-anesthetized rats (*n* = 5 control rats, *n* = 4 epileptic rats). PV basket cells were neurochemically identified in one of five (control) and one of four (epileptic) interneurons. They were immunoreactive to PV but not to somatostatin and had several synaptic boutons around pyramidal cell bodies (Fig. 7*H*). Their firing dynamic during theta and SPW ripples was consistent with previous juxtacellular data from control rats under urethane (Fig. 7*I*; Somogyi and Klausberger 2005), giving support to our tetrode classification in the TLE context.

A total of four putative PV basket cells were unequivocally identified in tetrodes from TLE animals (Table 1). Other interneuronal types were also recognized, but sample size prevented comparisons. Importantly, except for one control unit all putative PV basket cells were from proximal locations, allowing for direct comparisons between groups (Table 1). Group differences in the phase vector length and autocorrelation index were stronger for putative PV basket cells than for all interneurons (Fig. 7*J;* Table 1). Strikingly, a gamma autocorrelation index (40–90 Hz) was significantly lower in epileptic PV basket cells (Student’s *t* test, *p* = 0.0115; Fig. 7*J*) but not when all interneurons were considered (*p* = 0.2048; Table 1), supporting the idea that poor rhythmic firing of this specific interneuronal population may explain the disruption of slow gamma coupling to theta in the epileptic hippocampus.

## Discussion

Our data identify a cognitive link for theta–gamma disruption in TLE rats, pointing to a microcircuit rhythmopathy in periods free of seizures and/or epileptiform activities. We found that layer-specific, theta-nested, slow gamma (30–60 Hz) oscillations are attenuated in the proximal CA1 region of epileptic rats. Strikingly, poorly modulated slow gamma oscillations at SR explain part of the variability of spatial memory abilities in an episodic-like memory task. The identification of putative GABAergic interneurons, and PV basket cells in particular, suggest that the disruption of their firing at the falling theta phase may be at the basis of this rhythmopathy. Thus, our data suggest that in periods free of interictal and ictal activities, cognitive comorbidities of TLE may be intimately related to specific microcircuit dysfunction in this neurological condition.

Gamma oscillations are supposed to emerge locally from either interneuron–interneuron (ING models) or pyramidal cell–interneuron interactions (PING models; Bartos et al., 2007; Buzsáki and Wang, 2012). Presumably, inputs that are segregated across theta phases engage different sets of local interneurons, bringing each gamma generator to motion. Layer III entorhinal inputs at SLM are in phase with the firing of CCK basket and axo-axonic cells (Somogyi and Klausberger, 2005; Schomburg et al., 2014), which may drive a mid-gamma generator (60–90 Hz) at the theta peak. Whether these cell types are better activated by entorhinal inputs is unknown, but their preferential perisomatic targeting of PCs is at odds with the mid-gamma band isolated at the SLM sink (Fig. 3*A*; Lasztóczi and Klausberger, 2014; Schomburg et al., 2014). Possibly, perforant path-associated interneurons may play a role in this dendritic mid-gamma oscillation, either in response to layer II island cells (Sun et al., 2015) or due to entorhinal cortex long-range inhibition (Basu et al., 2016). Feedback disinhibition of PV-positive interneurons, including bistratified and axo-axonic cells, appears also necessary for mid-gamma oscillations at the theta peak (Wulff et al., 2009). The basic mechanisms of mid-gamma oscillations are still unclear.

We found that the theta peak-associated mid-gamma generator was attenuated in proximal SLM layers of TLE rats but was similarly modulated by amplitude compared with control rats (Fig. 3*D*,*E*). This possibly reflects a lower theta power reported in TLE rats (Inostroza et al., 2013) due to stronger sclerosis of layer III medial entorhinal neurons giving raise to the proximal temporoammonic pathway (Laurent et al., 2015). Changes in intrinsic and synaptic integration properties of OLM interneurons have been previously related to impaired theta rhythmogenesis in TLE with intact gamma oscillations (Dugladze et al., 2007). Interestingly, entorhinal cells are more excitable in the epileptic temporal lobe (Kobayashi et al., 2003; Tolner et al., 2007), suggesting that the modulation of perforant path-associated interneurons should still be in place even though their proper recruitment may be compromised (Morin et al., 1998). Thus, attenuated mid-gamma oscillations (60–90 Hz) at the proximal CA1 may be reflecting local changes occurring upstream at layer II and III of the medial entorhinal cortex (Laurent et al., 2015).

More critically, the proximal slow SR gamma generator (30–60 Hz) at the falling theta phase was reduced and poorly modulated in TLE animals (Fig. 3*E*,*F*). Recently, Schomburg et al. (2014) showed that local hippocampal theta–gamma coupling at 30–60 Hz is more prominent at the proximal CA1, where GABAergic interneurons are strongly driven by Schaffer collaterals (Oliva et al., 2016). Both in epileptic and control rats, slow gamma oscillations were in phase with CSD sinks at the SR, which is consistent with this feedforward activation model (Mann et al., 2005; Zemankovics et al., 2013; Butler et al., 2016). PV basket cell firing at the falling theta phase have been involved in the mechanism (Lasztóczi and Klausberger, 2014). This interneuronal population represents a relatively resilient cell type in the CA1 epileptic hippocampus that innervates surviving CA1 PCs (Wittner et al., 2005; Wyeth et al., 2010). We found that theta and gamma rhythmicity of PV basket cells was strongly compromised in TLE (Fig. 7*J*), and that this is likely to explain theta–gamma rhythmopathy in the slow-frequency band at the CA1 proximal pole.

Poor rhythmicity of PV basket cells during theta oscillations may result from many factors. Reduced synaptic recruitment of specific classes of interneurons is reported in TLE (Morin et al., 1998), suggesting that some of them may be failing to fire in response to timely inputs. Timely recruitment of PV basket cells determine coordinated oscillations and spatial representation (Korotkova et al., 2010). Interestingly, we noted a nonsignificant decline of the SPW/ripple ratio in PV basket cells of TLE rats (Table 1), pointing to their ineffective activation by Schaffer collaterals, but this could also be reflecting a sorting collapse during fast ripples (Ibarz et al., 2010; Merricks et al., 2015). Actually, enhancement of the fast gamma band (90–140 Hz) in TLE rats supports this view (Fig. 2*E*). While CA3 PCs are relatively preserved in this TLE model (Inostroza et al., 2011), reduced Schaffer inputs could still fail to recruit CA1 interneurons (Ang et al., 2006; Fig. 3*F*). Indeed, fluctuations of the CA1 slow SR gamma power parallels the mean firing rate of upstream CA3 PCs during sleep/run-associated theta and novelty (Mizuseki et al., 2009; Schomburg et al., 2014). Changes of intrinsic excitability in PV fast-spiking cells due to deficits of Nrg1-Erbb4 signaling reported in TLE could also explain our data (Li et al., 2011). Epileptic PV basket cells are particularly susceptible to entering the depolarizing block (Karlócai et al., 2014; Yi et al., 2015), but our intracellular recordings of identified cells suggest that this is not likely to be the case (Fig. 7*H*,*I*). Theta rhythmicity of PV basket cells may result in part from septal inputs (Hangya et al., 2009), which are affected in TLE (Colom et al., 2006). Thus, the exact mechanism leading to CA1 PV basket cell dysfunction in TLE is rather complex, requiring an independent investigation. Our data bring the focus to the interneuronal population, and PV basket cells specifically, as a key element of microcircuit rhythmopathies in the epileptic hippocampus.

We found that variability in theta phase modulation of the slow gamma (30–60 Hz) correlates with spatial memory deficits of epileptic rats in the episodic-like memory task (Fig. *4E*). This links theta–gamma coupling to episodic memory function and dysfunction, as demonstrated in humans (Lega et al., 2015, 2016). Importantly, our approach allows us to weight the role of oscillatory disruption over effects caused by seizures or epileptiform activity, giving support to the role of hippocampal rhythmopathies in cognitive comorbidities. Our data are consistent with the view that the depth to which theta modulates gamma power predicts memory for recent spatial episodes (Shirvalkar et al., 2010; Chang and Huerta, 2012; Kemere et al., 2013; Trimper et al., 2014). In contrast, coordination between entorhinal and hippocampal regions appears instrumental for temporal aspects of episodic memory (Suh et al., 2011; Inostroza et al., 2013; Laurent et al., 2015). Gamma oscillations and their theta phase preference adjust on demand, reflecting the balance of input activity along the entorhinal–hippocampal circuitry (Montgomery and Buzsáki, 2007; Schomburg et al., 2014). This dynamic ability reinforces CA3-CA1 interactions during novelty (Lever et al., 2010; Trimper et al., 2014), adjusts information flow from CA3 over the course of encoding and retrieval (Kunec et al., 2005; Douchamps et al., 2013), and organizes sequences of location across slow gamma phases (Zheng et al., 2016), all of which may be impaired in the epileptic hippocampus. Still, the specific role of theta and gamma in spatial and temporal memory is possibly far more complex and does not support a reductionist view. For instance, theta rhythmicity appears dispensable for new spatial representation (Brandon et al., 2014), theta–gamma coupling in the slow band underlies temporal coding (Allen et al., 2016), and a fast but not a slow gamma link to spatial-based strategies requiring integration along entorhinal–hippocampal networks (Cabral et al., 2014).

Although our current understanding of the role of hippocampal oscillations in cognition and the mechanisms underlying is yet limited, data suggest there are strong links. We have identified a microcircuit rhythmopathy of theta–gamma coupling at the slow band in the proximal SR of TLE rats. Poor rhythmicity of PV basket cells appears to be at the basis of this impairment. In models of Alzheimer’s disease, a disease linked to early-onset temporal lobe epilepsy and reduced gamma activity alteration, the excitability of PV interneurons has been associated with similar cognitive deficits (Verret et al., 2012). We propose that cognitive comorbidities of temporal lobe diseases share common principles at the microcircuit level and that PV interneurons are a critical target.

## Synthesis

Reviewing Editor: György Buzsáki, New York University Decisions are customarily a result of the Reviewing Editor and the peer reviewers coming together and discussing their recommendations until a consensus is reached. When revisions are invited, a fact-based synthesis statement explaining their decision and outlining what is needed to prepare a revision will be listed below.

Two main physiological alterations in TLE rats are reported: diminished str. radiatum theta -nested slow gamma oscillations (that has being show before to reflect CA3 to CA1 input) and reduced theta rhythmicity in CA1 PV-basket like interneurons. They related these alterations with impaired performance in a spatial memory task. Importantly, physiological and behavioral alterations are not attributable to seizures or inter-ictal spikes, as in other related rodent models of epilepsy (e.g. Gelinas et al., 2016), but rather to impaired hippocampal oscillatory dynamics. The present report adds important evidence about the role of cross-frequency coupling as a key mechanism in normal and pathological cognitive function.

1-A striking observation of this work is that most prominent slow gamma and interneuron alterations in TLE rats occurred specifically at CA1 proximal sites. If a reduced CA3 to CA1 input is at the basis of the observed deficits, one can expect that this effect should affect the whole dorsal CA1 area, which is innervated by the same CA3 axons. There are at least two possible explanations to this findings but it would be beneficial that the authors extend more in the discussion on their own explanation for this regional specificity. Schomburg et al., 2014 reported that theta nested slow gamma oscillation are more prominent in proximal sites, perhaps explaining why alterations in this gamma generator are more prominent there. Also, a recent report (Oliva et al., 2016, Hipp) showed stronger drive of proximal CA1 interneurons by CA3 pyramidals. In addition, as the authors already noted, medial (MEC) and lateral (LEC) entorhinal cortex project preferentially to proximal or distal CA1 poles. Several differences in physiological properties of CA1 neurons have been attributed to this differential innervation (Henrikssen et al., 2010; Hargreaves et al., 2005). Are the authors aware of some evidence that point to specific MEC but not LEC alteration in this model of TLE? The authors should at least mention this anatomical pattern in the discussion. Perhaps a reduced recruitment of local interneurons by both CA3 and MEC layer 3 inputs can explain the present results. To check gamma phase coupling of interneurons by both gammaS and gammaM could shed further light in this question.

2-Gamma oscillation in Fig 2A will be more clearly appreciable reducing the time shown and expanding the traces.

3-CA1 gamma components in Fig 2C are not clearly visible because the color scale is normalized to the larger hilar power. A more saturated color scale will make the plot clearer.

4-Although different theta-coupled gamma component are clearly appreciated in Fig. 3, the ideal approach to demonstrate the existence of cross-frequency coupling will be to perform a surrogate test on the phase-amplitude time series before the calculation of the modulation index.

5-In the calculation of theta-gamma modulation indices during the memory task (Fig 4 D-E), where only theta epochs included or the whole session? In the later case, the variable presence of theta oscillations (i.e. immobility periods) could be an important confounding factor

6-The reference to Lasztoczi and Klausberber 2014 is misspelled at many locations (line 673, 727, 733, 806, 832)

7-Fig 4A: It would increase the clarity of the behavioral task, if beside the schematic representation of the task the timeline appeared on the panel. The colors of the 'test' arrangement are confusing.

8-The legend of Fig 5D, 6C and 6F should be clarified.

9-In Fig 6A,B the crosses and ticks to represent spikes are redundant. The later can be removed.

10-For Fig 6E it would be better to show an example of PV-basket cell instead of an unclassified interneuron.

11. In the 1st paragraph of introduction cite Bragin et al., 1995, who first described theta-gamma coupling in hippocampus.

Methods

12. page 5: line 130

"All experiments started 8 weeks after status when rats already exhibited recurrent seizures"

13. How detection of seizures was performed?

In line 141-142 authors contradict themselves writing that "epileptic and saline-treated rats were implanted 6 weeks after treatment (please correct).

14. Page 15 lines 525-536: It is not clear if different frequency bands did not follow the 1/f law in all recording sites or only in SLM?

Fig. 1: abbreviation for SLM should be moved on the 4th line of the figure legend text.

Fig. 4: It is not clear what is the difference between epileptic group and epileptic with SZ/IS. Was there a group of epileptic rats without seizures?

It is related to my previous question. How epileptic and non-epileptic rats were distinguished? What percentage of rats became epileptic.
